# TorsinA rescues ER-associated stress and locomotive defects in *C. elegans* models of ALS

**DOI:** 10.1242/dmm.013615

**Published:** 2013-12-05

**Authors:** Michelle L. Thompson, Pan Chen, Xiaohui Yan, Hanna Kim, Akeem R. Borom, Nathan B. Roberts, Kim A. Caldwell, Guy A. Caldwell

**Affiliations:** 1 Department of Biological Sciences, The University of Alabama, Tuscaloosa, AL 35487, USA.; 2 Departments of Neurology and Neurobiology, Center for Neurodegeneration and Experimental Therapeutics, Gregory Fleming James Cystic Fibrosis Research Center, University of Alabama at Birmingham, Birmingham, AL 35294, USA.

**Keywords:** ALS, ER stress, Chaperone, Neurotransmission, TorsinA

## Abstract

Molecular mechanisms underlying neurodegenerative diseases converge at the interface of pathways impacting cellular stress, protein homeostasis and aging. Targeting the intrinsic capacities of neuroprotective proteins to restore neuronal function and/or attenuate degeneration represents a potential means toward therapeutic intervention. The product of the human *DYT1* gene, torsinA, is a member of the functionally diverse AAA+ family of proteins and exhibits robust molecular-chaperone-like activity, both *in vitro* and *in vivo*. Although mutations in *DYT1* are associated with a rare form of heritable generalized dystonia, the native function of torsinA seems to be cytoprotective in maintaining the cellular threshold to endoplasmic reticulum (ER) stress. Here we explore the potential for torsinA to serve as a buffer to attenuate the cellular consequences of misfolded-protein stress as it pertains to the neurodegenerative disease amyotrophic lateral sclerosis (ALS). The selective vulnerability of motor neurons to degeneration in ALS mouse models harboring mutations in superoxide dismutase (SOD1) has been found to correlate with regional-specific ER stress in brains. Using *Caenorhabditis elegans* as a system to model ER stress, we generated transgenic nematodes overexpressing either wild-type or mutant human SOD1 to evaluate their relative impact on ER stress induction *in vivo*. These studies revealed a mutant-SOD1-specific increase in ER stress that was further exacerbated by changes in temperature, all of which was robustly attenuated by co-expression of torsinA. Moreover, through complementary behavioral analysis, torsinA was able to restore normal neuronal function in mutant G85R SOD1 animals. Furthermore, torsinA targeted mutant SOD1 for degradation via the proteasome, representing mechanistic insight on the activity that torsinA has on aggregate-prone proteins. These results expand our understanding of proteostatic mechanisms influencing neuronal dysfunction in ALS, while simultaneously highlighting the potential for torsinA as a novel target for therapeutic development.

## INTRODUCTION

Amyotrophic lateral sclerosis (ALS) is a neurodegenerative disorder that affects the upper and lower motor neurons; it provokes muscle weakness that initiates in the extremities, eventually disrupting muscles controlling respiration. Patients suffer from paralysis and death due to respiratory failure within 1–5 years of diagnosis (Cleveland and Rothstein, 2001). There are various proteins linked to ALS, including, but not limited to, TDP-43, SETX and VCP, which influence pathways mediating RNA metabolism, helicase activity and proteasome activity, respectively (Pasinelli and Brown, 2006).

Although there is similarity in function among a few proteins associated with ALS (TDP-43, FUS, ATXN2), like most neurodegenerative diseases, multiple factors (genetic and environmental) and pathways lead to motor neuron decline (Ferraiuolo et al., 2011; Pasinelli and Brown, 2006). These factors disrupt key protein interactions due to protein misfolding, decreased chaperone activity, altered axonal transport and dysfunctional mitochondrial metabolism (Pasinelli and Brown, 2006). Because many of the proteins associated with ALS are ubiquitous in expression, the sensitivity of motor neurons to alterations in environment or protein-folding state is still in question.

Studies have shown that individuals suffering from ALS, and models that recapitulate the disease, have higher levels of endoplasmic reticulum (ER) stress in their motor neurons when compared with controls. Autopsied tissues and spinal cords from ALS patients, as well as ALS mice, have all been shown to exhibit an upregulation of ER stress proteins (Ilieva et al., 2007; Kikuchi et al., 2006). Furthermore, a subclass of motor neurons linked to familial ALS is more vulnerable to ER stress and contributes to the rapid progression of the disease (Saxena et al., 2009).

A dynamic factor contributing to ER stress is oxidative stress. The large cell size of motor neurons, a physical factor associated with ALS susceptibility, places a great demand on the mitochondria for energy metabolism (Kanekura et al., 2009). With a genetic predisposition due to natural variations in genetic code or specific, inherited genetic mutations linked to a disease, oxidative stress can contribute to physiological strain. This is especially the case when there is any perturbation to mitochondria or components that maintain homeostasis. In conjunction, the oxidative rich environment of the ER assists in proper protein disulfide bond formation yet permits this precarious cycle of potential stress to continue. This imbalance can contribute to ER stress; when prolonged, induction of cellular death pathways occurs (Ferraiuolo et al., 2011; Malhotra and Kaufman, 2007). Interestingly, a mutation in vesicle-associated membrane protein-associated protein B (VAPB), an ER-associated transmembrane protein that has been shown to interact with a key ER stress regulator, ATF6, has been identified as the familial ALS gene, *ALS8* (Gkogkas et al., 2008; Nishimura et al., 2004).

**TRANSLATIONAL IMPACT**

**Clinical issue**

The clinical pathology of amyotrophic lateral sclerosis (ALS) includes the selective degeneration of both upper and lower motor neurons in the spinal cord and cerebral cortex that can originate from either genetic or sporadic causes. Irrespective of causality, one of the crucial outlying questions in ALS research is centered on why specific motor neurons succumb to pathological demise in response to mutant proteins linked to the disease – a phenomenon known as selective vulnerability. Heritable mutations in the gene encoding superoxide dismutase 1 (SOD1) represent a well-studied contributor to ALS pathogenesis. Studies on animal models expressing mutant SOD1 have provided evidence that the subsets of neurons that exhibit selective vulnerability in brains are more prone to increased stress associated with protein misfolding at the endoplasmic reticulum (ER).

**Results**

The authors take advantage of both established and novel *Caenorhabditis elegans* models to evaluate the consequences of mutant SOD1 on cellular stress and neurological function. In worms, mutant SOD1 protein expression recapitulates key molecular features of ALS, including loss of motor neuron control and induction of ER stress by misfolded SOD1. Prior research revealed that torsinA, a chaperone-like protein expressed highly in the brain, can function as a molecular buffer against cellular stress associated with protein misfolding. Using *C. elegans*, the authors provide multiple lines of evidence showing that increased torsinA levels attenuate stress caused by mutant SOD1 and also rescue a locomotion defect associated with motor neuron dysfunction. RNAi and mutant analysis provided evidence that the mechanism of torsinA-mediated protection involves the ER protein-degradation component Derlin-1.

**Implications and future directions**

*C. elegans* enables highly selective quantitative analysis across genetically invariant animal populations with statistical confidence to evaluate genetic and chemical modifiers of disease-associated readouts. The outcomes of this study indicate that torsinA can serve as a functional modifier of mutant SOD1, with the capacity to manage intracellular stress and restore levels of neuronal function *in vivo*. These data corroborate prior studies that suggested a role for ER stress in ALS, and also extend our understanding of the range of torsinA activity in maintaining proteostasis. In this context, it is significant that small-molecule enhancers of torsinA activity have been reported (in *Disease Models & Mechanisms*) and represent putative molecular leads for ALS therapeutic development. This study provides a framework for further investigation into the role of ER stress in ALS pathophysiology, particularly as it has emerged as an important factor in models involving other ALS-related proteins, including TDP-43 and FUS.

One of the most studied mutant proteins associated with familial ALS cases is the Cu/Zn superoxide dismutase protein, SOD1. This key regulatory enzyme is needed to maintain homeostasis of superoxide radicals in the body; it converts superoxide radicals into molecular oxygen and hydrogen peroxide (Fridovich, 1986). The pathogenic variations within the enzyme subunit result in an unstable protein, causing the protein to misfold and form insoluble aggregates (Hart, 2006). Surprisingly, ALS occurs whether the mutant forms have dismutase activity or not, indicating that SOD1 toxicity is mediated through gain-of-function properties (Wang et al., 2009). This conclusion is supported by data obtained from SOD1-knockout mouse experiments, because these animals do not develop ALS (Shefner et al., 1999). Regulators of antioxidation have been shown to be dysregulated in mutant SOD1 models and patients (Kirby et al., 2005; Sarlette et al., 2008). Moreover, various forms of mutant SOD1 accumulate in the mitochondria, allowing aberrant chemistry such as peroxidation, tyrosine nitration and reverse catalysis to occur (Pasinelli and Brown, 2006).

The expression of SOD1 in the cytoplasm of multiple cell types supports a critical role for this protein in maintaining homeostasis. Because mutant SOD1 misfolds, homeostasis is disrupted, inducing oxidative and ER stress (Atkin et al., 2006). Cells manage misfolded proteins through a process termed the unfolded protein response (UPR). This mechanism initially involves shuttling misfolded proteins back to the ER for proper refolding to occur with the aid of molecular chaperones. However, if the protein is terminally misfolded, a secondary response is to transport these proteins to the cytosol, which contains all the catalytic enzymes necessary to selectively target (i.e. via ubiquitylation machinery) the misfolded proteins for proteasomal degradation (Meusser et al., 2005). This latter process is termed ER-associated degradation (ERAD). Proteins that accumulate during ERAD trigger the UPR and, if the cell cannot ameliorate this, it will undergo apoptosis.

The cellular machinery transports misfolded proteins from the ER lumen to the cytoplasm by shuttling them through the retrotranslocon complex. This complex is comprised of multiple proteins, including Derlin-1, VIMP, p97, Sec61 and Hrd1 E3 ligase (Schulze et al., 2005; Urushitani et al., 2004). It has been shown that mutant SOD1, not the wild-type (WT) form, induces ER stress in a Derlin-1-dependent manner. The exact mechanism by which mutant SOD1 eludes ERAD remains unknown but it has been hypothesized to involve interfering with the final transfer of ubiquitin to the misfolded protein by the E3 ligase (Le Pecheur et al., 2005; Nishitoh et al., 2008). Overall, when ERAD is blocked, ER stress is induced and activation of the ROS-apoptotic ASK1-p38 pathway occurs, leading to cellular toxicity (Urushitani et al., 2004; Wengenack et al., 2004). When Derlin-1 is overexpressed, thereby promoting proteasomal activity, mutant SOD1 accumulation is reduced and ER stress is restored to basal levels (Mori et al., 2011; Nishitoh et al., 2008; Urushitani et al., 2002; Urushitani et al., 2004).

In the ER lumen, the targeted substrates are retained by molecular chaperones in order for them to be transferred to the retrotranslocon complex for proper transfer to the proteasome (Nakatsukasa and Brodsky, 2008). Chaperones, including BiP (i.e. Hsp70/GRP78) and Hsp40, have been shown to prevent soluble aggregates of protein from forming prior to degradation (Nishikawa et al., 2001). BiP is also an established regulator of ER stress transducers. In this context, another key chaperone protein of interest is torsinA. In a mutant form, this protein causes a neurological movement disorder, early-onset torsion dystonia (DYT1), and mislocalizes to the nuclear envelope (NE). In WT form, torsinA is primarily localized to the ER lumen (Liu et al., 2003). We, as well as others, have shown in multiple systems that torsinA acts as a mediator of ER stress, in that it has the ability to attenuate protein aggregation and, most recently, it has been demonstrated to promote ERAD of terminally misfolded proteins (Chen et al., 2010; Hamamichi et al., 2008; Nery et al., 2011). As an AAA+ ATPase protein, torsinA exhibits robust chaperone activity and is hypothesized to assemble into oligomeric complexes that undergo conformational changes with coupled ATP-binding and -hydrolysis reactions (Chen et al., 2010; Naismith et al., 2004).

Most notably, torsinA was recently found to associate with ERAD components, including Derlin-1, VIMP and p97, and promote the degradation of mutant CFTR and cholera toxin (Nery et al., 2011). Therefore, we hypothesized that torsinA has the capacity to rescue ER stress induced by mutant SOD1 and promote proper ERAD. To test this hypothesis, we turned to a well-established *Caenorhabditis elegans* model of ALS (Wang et al., 2009). In these nematodes, human WT and mutant SOD1 are overexpressed pan-neuronally; the mutant SOD1 forms insoluble aggregates and induces neurotransmission defects resulting in locomotive dysfunction (Wang et al., 2009). These neurotransmission defects have been linked to a presynaptic deficiency where a significant decrease in the number of synaptic vesicles was observed. Thus, an association was established between SOD1 toxicity and disrupted protein biogenesis and trafficking, within the context of a readily scored behavioral phenotype.

We utilized these transgenic nematodes to investigate the impact of human torsinA expression on SOD1 cellular dynamics. These studies demonstrated that torsinA was able to rescue the presynaptic defect caused by human G85R SOD1 *in vivo*, and that rescue of the behavioral defect was mediated by Derlin-1. Furthermore, we examined the impact of SOD1 mutant G85R using a sensitive *in vivo* ER stress assay in transgenic nematodes (Chen et al., 2010; Nery et al., 2011). Alone, G85R SOD1 enhanced the ER stress response in *C. elegans*; however, co-overexpression of torsinA attenuated this effect.

## RESULTS

### TorsinA rescues G85R-SOD1-induced ER stress

We wanted to determine whether mutant SOD1 would induce an ER stress response in *C. elegans* using the well-characterized transgenic ER stress reporter strain *hsp-4*::GFP (Chen et al., 2010; Nery et al., 2011). *C. elegans* HSP-4 is homologous to the mammalian ER-resident chaperone BiP (Calfon et al., 2002). When the *hsp-4* promoter is engineered to drive GFP expression, this provides a quantitative measure of the induction of ER stress response. We expressed human WT SOD1 and G85R SOD1 under the *C. elegans* intestine-specific promoter *ges-1* (P*_ges-1_*::WT SOD-1; P*_ges-1_*::G85R SOD1). These constructs were injected independently into nematodes expressing an integrated *hsp-4*::GFP transgene. Following the production of stable transgenic lines of animals, GFP pixel intensities were assayed for each construct generated.

**Fig. 1.**
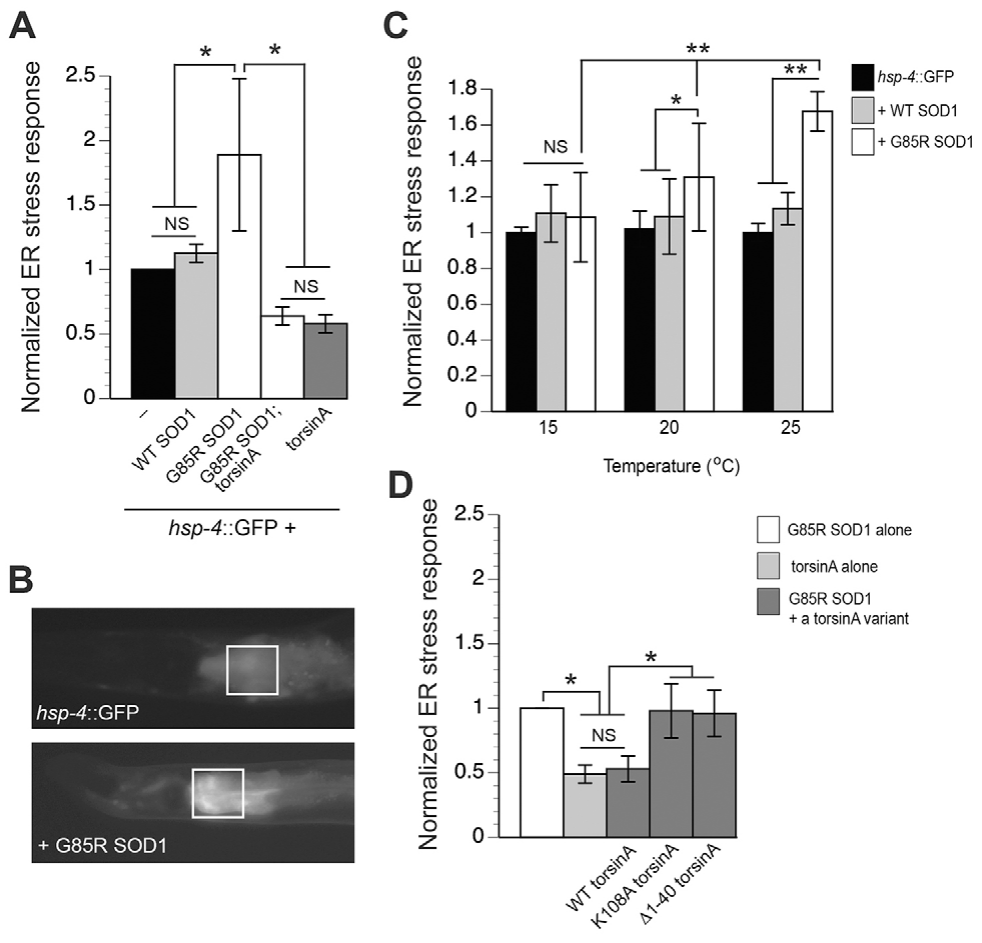
**TorsinA overexpression restores ER homeostasis to animals expressing G85R SOD1.** (A) Bar chart representing normalized GFP pixel intensity values measured consistently in the boxed region shown in B. These worms express the *hsp-4*::GFP reporter alone or with other transgenic constructs as displayed in the figure. Values are the mean ± s.d. of three experiments where 30 animals were analyzed per replicate (*P<0.01; Student’s *t*-test). Animals expressing G85R SOD1 elicit an ER stress response, whereas WT SOD1 animals display an ER stress response that is comparable to *hsp-4*::GFP animals. (B) Representative fluorescent micrographs of animals expressing the ER stress reporter *hsp-4*::GFP alone (upper panel) and with an intestinally expressed G85R *SOD1* transgene (lower panel). The image depicts the anterior region of *C. elegans* and the boxed region indicates the 100×100 μm region just below the pharynx where measurements of fluorescence were taken in all animals. (C) The stress response of animals expressing G85R SOD1 was temperature dependent. The relative stress level of G85R SOD1 worms increased significantly at 20°C and 25°C compared with the response at 15°C (**P<0.001; Student’s *t*-test). Furthermore, worms expressing G85R SOD1 also displayed significantly more ER stress compared with those expressing either WT SOD1 or the *hsp-4*::GFP control at 20°C and 25°C, confirming a physiological temperature-sensitive characteristic of the mutant G85R SOD1 protein (*P<0.01; **P<0.001; Student’s *t*-test). (D) Bar chart representing normalized GFP pixel intensity values. These worms express the *hsp-4*::GFP reporter alone or with other transgenic constructs as displayed in the figure. Values are the mean ± s.d. of three experiments where 30 animals were analyzed per replicate (*P<0.05; one-way ANOVA analysis with Tukey post-hoc test). Animals expressing G85R SOD1 elicit an ER stress response that is not restored by torsinA variants K108A and Δ1–40 torsinA, as seen with WT torsinA, where there is a significant reduction in ER stress response. All normalized values are to G85R SOD1 fluorescent intensities.

WT SOD1 worms displayed a stress level comparable to control (*hsp-4*::GFP only) worms, whereas G85R SOD1 animals exhibited severe ER stress [~2-fold induction (*P*<0.02; Fig. 1A,B)]. A previous mammalian cell-based study demonstrated that G85R SOD1 caused enhanced ER stress (Le Pecheur et al., 2005) and our *in vivo* model recapitulated these results. In this regard, our data provide evidence for a conserved mutant SOD1-associated ER stress response among various metazoans.

Previous cell culture studies have shown that SOD1 metal-binding mutants, such as G85R SOD1, are sensitive to temperature change (Rodriguez et al., 2002; Tiwari and Hayward, 2005) and these alterations can influence protein stability. To determine whether our ER stress model using this same G85R mutation reflected this temperature-sensitive property of SOD1, we characterized the ER stress response using a temperature gradient. We chose three temperatures (15°C, 20°C and 25°C) that are amenable to the growth of *C. elegans*. In transgenic strains that expressed *hsp-4*::GFP only, or with WT SOD1, the elevated temperatures did not modify the ER stress response. In contrast, we found that the ER stress response induced by G85R SOD1 was statistically enhanced with higher temperatures (*P*<0.001; Fig. 1C). These data are consistent with the temperature-sensitive property previously described for G85R SOD1 in cell culture (Rodriguez et al., 2002; Tiwari and Hayward, 2005).

To determine whether torsinA could modify the ER stress response caused by G85R SOD1, these animals were crossed with a transgenic line expressing human torsinA specifically within the intestine (P*_ges-1_*::torsinA) (Chen et al., 2010). Animals were grown at an intermediate temperature, 20°C, as described previously (Fig. 1A). TorsinA animals significantly reduced the basal level of ER stress response when compared with *hsp-4*::GFP animals alone (*P*<0.01). The ER stress response induced by G85R SOD1 was completely attenuated by torsinA (*P*<0.01; Fig. 1A). Notably, the level of ER stress response in animals that expressed torsinA alone and those that expressed torsinA + G85R SOD1 was not significantly different, reflecting a restoration of ER homeostasis. Thus, torsinA functions as a potent modulator of ER stress, and it abolishes the ER stress associated with G85R SOD1.

To determine whether the rescue of the ER stress response observed was dependent on torsinA activity, we overexpressed two torsinA variants in the G85R SOD1 mutant background. These are well-studied torsinA variants that abolish torsinA activity in all functional assays examined in mammalian and human cells, as well as *C. elegans* assays. Among these is the K108A mutation, which prevents ATP hydrolysis but retains ER localization (Goodchild and Dauer, 2004; Naismith et al., 2004). The second variant consisted of Δ1–40 torsinA, which eliminates torsinA retention to the ER (Liu et al., 2003). Our lab has previously published that both these variants eliminate the ability of torsinA to rescue the ER stress response (Chen et al., 2010). As independent constructs, we overexpressed K108A torsinA and Δ1–40 torsinA in combination with G85R SOD1 and found that, unlike WT torsinA, neither torsinA variant was able to attenuate the ER stress response induced by mutant SOD1 (Fig. 1D). Thus, this provided evidence that the cytoprotective effect observed was WT-torsinA-specific and requires ATPase activity, as well as ER localization (Chen et al., 2010; Liu et al., 2003).

### TorsinA attenuates the neurotransmission defects of G85R SOD1 animals

**Fig. 2.**
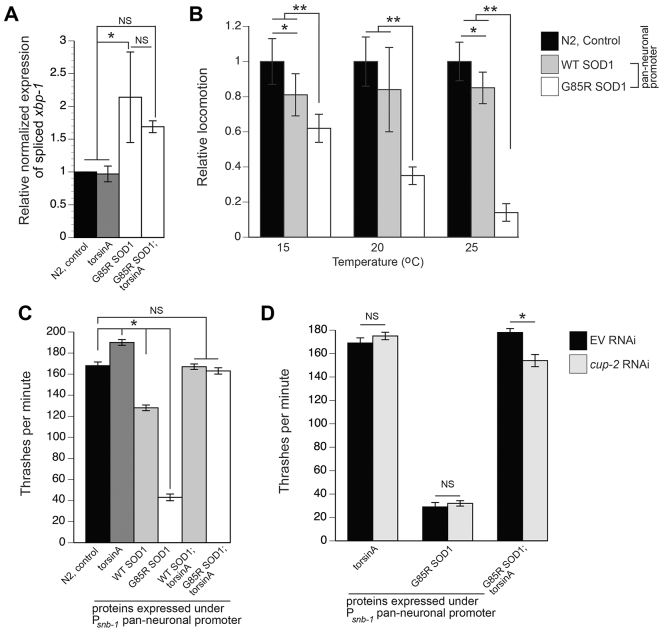
**TorsinA rescue of G85R SOD1 thrashing defect is partially dependent on Derlin-1.** (A) Nematodes expressing G85R SOD1 pan-neuronally induce ER stress, represented through elevated alternatively spliced *xbp-1* transcript levels, measured through quantitative PCR (qPCR). When co-expressed with torsinA, G85R SOD1 ER stress is reduced slightly, albeit non-significantly. Data are represented as normalized expression of spliced *xbp-1* (*P<0.05; one-way ANOVA followed by a Tukey post-hoc test). (B) Nematodes expressing G85R SOD1 pan-neuronally exhibit a temperature-dependent locomotion response on solid media. The most severe defect is observed when animals are cultivated and analyzed at 25°C, although at all temperatures G85R SOD1 animals exhibit a significant locomotion defect. WT SOD1 animals also exhibit locomotion defects, at 15°C and 25°C, compared with control animals that do not express SOD1 (*P<0.05; **P<0.001), but this effect is not temperature dependent. (C) Liquid thrashing analysis confirmed the locomotion defects of both WT- and G85R-SOD1-expressing animals (*P<0.05). TorsinA co-expression fully restored the aberrant thrashing defects of both WT SOD1 and G85R SOD1 animals to levels comparable to N2 control animals. (D) TorsinA-mediated rescue of G85R SOD1 in the liquid thrashing assay is dependent on the *C. elegans* Derlin-1 homolog, *cup-2*. Knocking down *cup-2* had no effect on *C. elegans* thrashing rates for transgenic strains expressing torsinA or G85R alone compared with empty vector (EV) RNAi control. In contrast, when G85R SOD1;torsinA animals are treated with *cup-2* (RNAi), thrashing rates are significantly reduced (*P<0.05; Student’s *t*-test). Data reflect the thrashing rate of animals cultivated at 20°C. Error bars represent ± s.e.m. of three experiments. 30 animals were analyzed per replicate [NS, non-significant difference (Student’s *t*-test)].

Following the observation that WT torsinA could rescue ER stress induced by G85R SOD1, we wanted to examine the impact of torsinA restoring ER stress using an alternative readout. We therefore shifted our analysis to use a well-established *C. elegans* model of ALS that expressed G85R SOD1 under a pan-neuronal promoter (P*_snb-1_*::G85R SOD1) in which insoluble protein aggregates are formed and associated locomotion defects are observed (Wang et al., 2009). To corroborate our previous ER stress data with G85R animals (P*_ges-1_*::G85R SOD1 transgenics), we assayed these pan-neuronal-expressing animals to confirm the induction of an elevated ER stress response (Fig. 2A). Upon ER stress, the *xbp-1* transcript is alternatively spliced (Calfon et al., 2002; Shen et al., 2001). In the pan-neuronal-expressing G85R SOD1 worms, significantly increased spliced *xbp-1* levels were detected by quantitative RT-PCR and these were reduced slightly, although not significantly, with co-expression of torsinA (G85R;torsinA) (Fig. 2A). These data provide evidence of induced ER stress in animals expressing G85R SOD1.

In these pan-neuronally SOD1-expressing worms, a reduced locomotive behavioral phenotype is observed in mutant SOD1-expressing nematodes, whereas no defect was evident with WT-SOD1-expressing or control animals (Wang et al., 2009). In a similar manner to the temperature-sensitive nature of G85R SOD1 and ER stress response, we wanted to determine whether *C. elegans* that expressed G85R SOD1 displayed a temperature-sensitive locomotive phenotype. Therefore, we measured the locomotion of L4-staged worms using the same temperature gradient as was used in the ER stress assay (15°C, 20°C and 25°C). Animals were cultivated and analyzed on solid medium (NGM plates), as previously described (Wang et al., 2009). We determined that worms that expressed G85R SOD1 displayed a temperature-dependent locomotion response whereby they exhibited nearly complete paralysis at 25°C and showed improved locomotion with lower temperatures (Fig. 2B). At 15°C, the mutant animals had an ~4-fold increase in locomotion compared with locomotion at 25°C. Although this rate was more similar to the rate observed for WT SOD1 transgenic worms and control (N2 Bristol) animals, it was still significantly lower (*P*<0.001; Student’s *t*-test; Fig. 2B). Interestingly, at 15°C and 25°C, animals expressing WT SOD1 displayed a difference in locomotion when compared with control animals (N2). However, there was not a temperature-dependent response as observed with the G85R SOD1 animals. These data offer *in vivo* evidence that the pan-neuronal P*_snb-1_*::G85R SOD1 strain displays a temperature-sensitive phenotype. For all subsequent analyses, studies were performed on animals cultivated at 20°C.

Another quantitative measurement for examining locomotive defects in *C. elegans* involves liquid thrashing assays. Unlike the previously described results examining behavior on solid media, liquid thrashing assays are useful in detecting subtle deficiencies resulting from the constant motion in a liquid medium. The same pan-neuronal strains were placed in water and the thrashing rates monitored for 1 minute. As shown in Fig. 2C, G85R-SOD1-expressing animals have a severe defect when compared with control N2 and WT SOD1 animals. These data are consistent with the locomotion assay performed on plates (Fig. 2B). Interestingly, when compared with control N2 animals, WT SOD1 animals also showed a slight, but significant, liquid thrashing defect, supporting our findings with the locomotion assay performed on plates (Fig. 2B,C).

To investigate the impact of torsinA on the SOD1-associated locomotion defect, we used the same pan-neuronal promoter and generated worm strains that overexpressed torsinA, creating the P*_snb-1_*::torsinA strain. When WT SOD1 or G85R SOD1 animals co-expressed torsinA, the thrashing rates were restored to control N2 animal levels (Fig. 2C) in day-3 adult animals. Further thrashing analyses on all these transgenic animals were conducted at additional time points (L4 stage and 5-day-old adults); the ability of torsinA to restore normal movement in both WT- and G85R-SOD1-expressing animals was observed at all stages analyzed (data not shown).

Through RNAi analysis, reduced function in protein chaperones, protein turnover and modification enhanced the locomotion defects of G85R SOD1 animals (Wang et al., 2009). These results indicate that the proteasome mediates G85R SOD1 toxicity and chaperones assist in maintaining homeostasis. Mutant SOD1 was also shown to disrupt ERAD through dysfunction of the retrotranslocation complex in a Derlin-1-dependent manner (Nishitoh et al., 2008). In a separate cell culture study, torsinA associated with ERAD proteins, including Derlin-1 (Nery et al., 2011). Thus, we wanted to determine whether the torsinA-mediated rescue of the mutant SOD1 locomotive defect was associated with Derlin-1 function. We knocked down the worm homolog of Derlin-1, termed *cup-2*, by RNAi. We then tested the thrashing rates of animals expressing G85R SOD1 + torsinA, torsinA alone, and G85R SOD1 alone. Reducing *cup-2* levels in worms expressing torsinA or G85R SOD1 alone did not influence their thrashing rates (Fig. 2D). However, depletion of *cup-2* resulted in a significant reduction in the thrashing rate in animals co-expressing G85R SOD1 and torsinA (Fig. 2D). Although reducing *cup-2* levels did not completely abolish the thrashing rescue mediated by torsinA, these data suggest that these two proteins might work together to alleviate the toxicity associated with the mutant SOD1 protein. Thus, the protection might be dependent on both torsinA and CUP-2/Derlin-1 proteins involved in promoting degradation of mutant misfolded SOD1.

In addition to the behavioral phenotype associated with the pan-neuronal G85R SOD1 animals, Wang and colleagues discerned this locomotive defect to be associated with a presynaptic defect (Wang et al., 2009). Specifically, these animals exhibit a significant decrease in the number of synaptic vesicles in the DA motor neurons and morphological abnormalities of the ventral nerve cord without any morphological changes to the innervated body wall muscles. Through pharmacological exposure to the cholinesterase inhibitor aldicarb and a cholinergic receptor agonist, levamisole, they demonstrated that G85R-SOD1-expressing animals were resistant to aldicarb but not to levamisole. These researchers attributed the phenotype to a presynaptic defect and suggested the protein trafficking and/or biosynthesis of acetylcholine was disrupted (Wang et al., 2009).

**Fig. 3.**
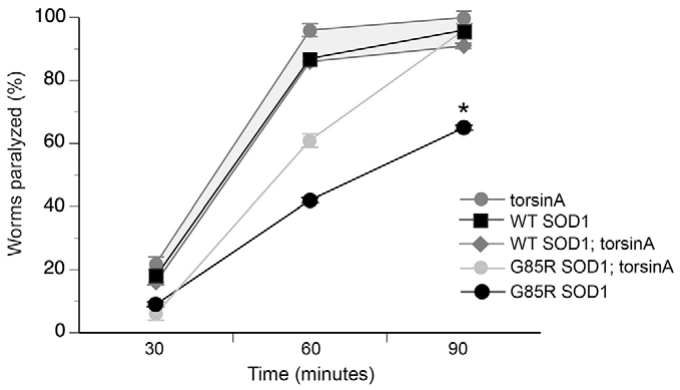
**TorsinA rescues the presynaptic defect associated with G85R SOD1 animals.** The percentage of animals paralyzed following exposure to 0.5 mM aldicarb for 90 minutes; analyses occurred every 30 minutes with 30 animals per replicate for a total of three replicates per transgenic line. G85R SOD1 animals were resistant to aldicarb after exposure to chemical for 60 and 90 minutes. TorsinA significantly rescued G85R SOD1 animals after 90 minutes compared with animals expressing G85R SOD1 alone (*P<0.05; Student’s *t*-test). Trends in sensitivity to aldicarb are highlighted with gray shading. Data reflect percent animals paralyzed when cultivated at 20°C. Error bars represent ± s.e.m. of three experiments.

To determine whether torsinA could attenuate the presynaptic defect defined in *C. elegans* that express G85R SOD1 pan-neuronally, assays were performed with these pharmacological modifiers on worms that co-expressed WT torsinA. After 90 minutes of exposure to aldicarb, 65% of the G85R SOD1 animals were paralyzed (Fig. 3) compared with 96% of animals that co-expressed torsinA with mutant SOD1 (*P*<0.05). Populations of worms expressing torsinA alone, WT SOD1 alone, or WT SOD1;torsinA displayed 100%, 96% or 91% paralysis after 90 minutes as well, respectively. These data support the previously published report of a synaptic defect when worms express G85R SOD1 pan-neuronally. Moreover, when torsinA was co-expressed, this G85R SOD1 synaptic deficiency was rescued *in vivo*.

### TorsinA reduces SOD1 protein levels

**Fig. 4.**
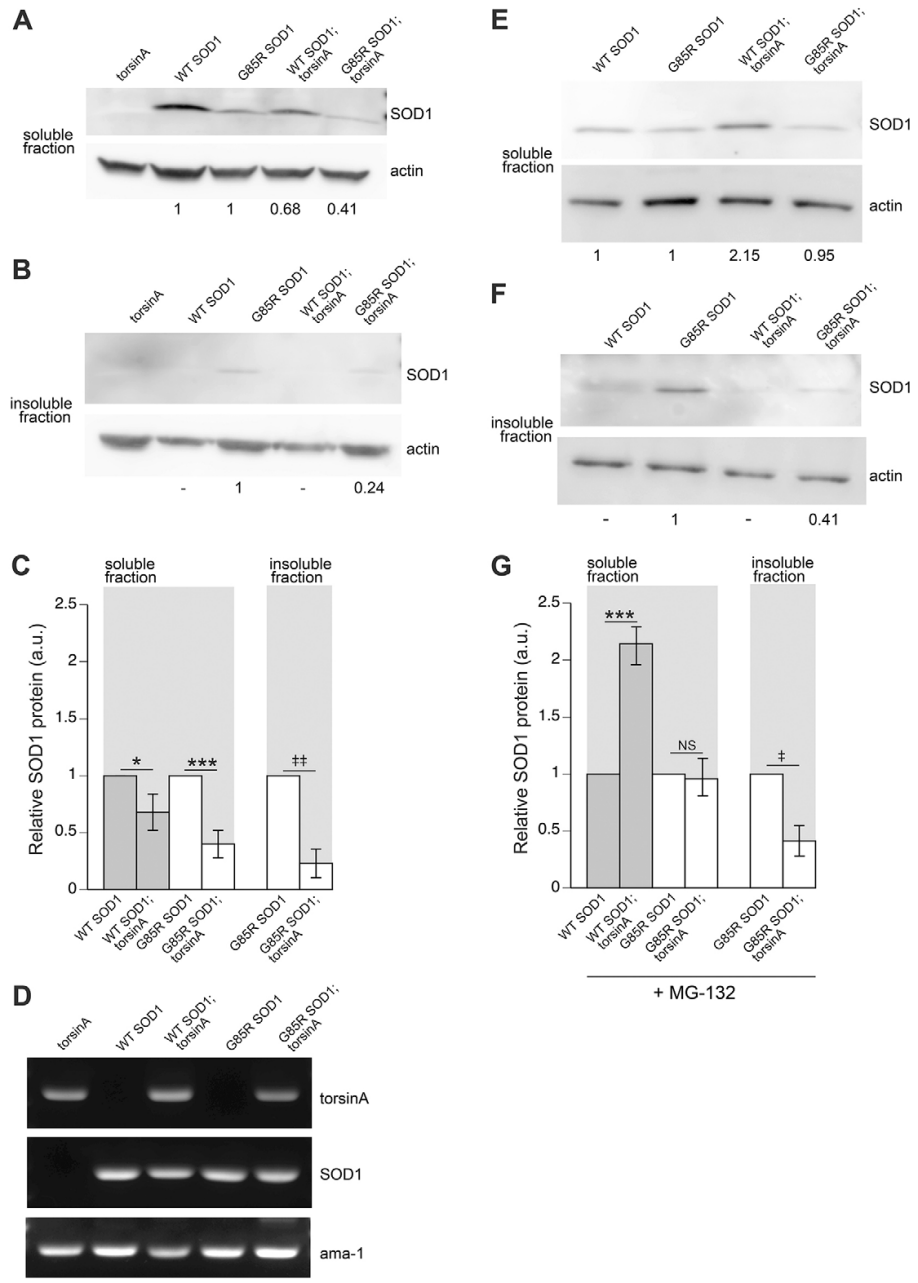
**TorsinA promotes degradation of SOD1 protein through the proteasome.** Representative western blots of protein isolated from *C. elegans* expressing torsinA and/or SOD1 from a pan-neuronal promoter with (E–G) or without (A–D) 50 μM MG-132 proteasome inhibitor treatment. These extracts were subjected to centrifugation to isolate soluble (A,E) and insoluble (B,F) fractions. These fractions were probed with antibody against human SOD1. An antibody to actin was used as a loading control. The normalized intensity values for SOD1 protein are listed below each blot image. Normalization was performed pair-wise with respect to SOD1 variant. For example, WT SOD1;torsinA was compared relative to WT SOD1 only and G85R SOD1;torsinA was normalized relative to G85R SOD1. Multigage software was used to gather quantitative data. (C,G) Graphical representation of three independent experiments. (C) The amount of total protein from animals expressing both G85R SOD1;torsinA was greatly reduced when compared with animals expressing G85R SOD1 alone. This was observed in both the supernatant (soluble) and pellet (insoluble) fractions and corresponded to an ~60% (***P<0.001) and ~80% (^‡‡^*P*<0.01) decrease in soluble and insoluble fractions. TorsinA also had a significant effect on WT SOD1 protein levels, demonstrating a 30% decrease in WT SOD1 soluble protein levels when torsinA is co-expressed (*P<0.05). (D) Semi-quantitative RT-PCR indicating that torsinA does not modify the mRNA levels of *SOD1* in any of the transgenic lines expressing torsinA. Primers specific for *torsinA* and *SOD1* were used to amplify ~200-bp products. Amplification of the housekeeping gene *ama-1* was used as a loading control. (G) A graphical representation of three independent experiments for animals treated with the MG-132 proteasome inhibitor. When torsinA is co-expressed with WT SOD1, the relative intensity values of WT SOD1 protein increased ~2-fold in the soluble fraction (***P<0.001). The G85R SOD1 protein levels are comparable, with and without torsinA co-expression in the soluble fraction. Conversely, G85R SOD1;torsinA protein levels are significantly lower compared with G85R SOD1 alone in the insoluble fraction (^‡^*P*<0.05). Error bars represent s.d. of three experiments (NS=non-significant; one-way ANOVA followed by a Tukey post-hoc test was utilized for soluble fraction comparisons, whereas a Student’s *t*-test was used for the insoluble fractions).

Previously published reports describe a role for torsinA as a regulator of protein homeostasis (Chen et al., 2010) and in promoting the degradation of misfolded proteins through ERAD (Nery et al., 2011). Therefore, we wanted to determine whether SOD1 protein levels were altered by the activity of torsinA. Using the transgenic nematode lines pan-neuronally overexpressing WT SOD1 and G85R SOD1, both with and without torsinA, the soluble and insoluble protein fractions from whole-animal extracts were examined. In the soluble fraction, the presence of torsinA caused an ~30% reduction in WT SOD1 protein levels (*P*<0.05). When torsinA was co-expressed with G85R SOD1 there was an ~60% decrease in protein levels in the soluble fraction (*P*<0.001) (Fig. 4A,C). G85R SOD1 in the insoluble fraction was decreased by ~75% (^‡‡^*P*<0.01) (Fig. 4B,C). The relative protein levels were determined for each respective protein (i.e. WT SOD1;torsinA was normalized to WT SOD1 and G85R SOD1;torsinA was normalized to G85R SOD1, along with respective actin control protein levels). TorsinA significantly reduced the relative amount of SOD1 protein in both soluble and insoluble fractions. Semi-quantitative RT-PCR was performed on cDNA isolated from these animals and there were no signs of mRNA downregulation in the presence of torsinA (Fig. 4D).

To determine whether the reduced SOD1 protein levels were due to torsinA promoting proteasomal degradation (i.e. ERAD), the pan-neuronal transgenic animals were cultivated in the presence of the proteasome inhibitor MG-132. Animals were exposed from the embryo to L4 larval stage, after which protein was extracted. There was a 2-fold increase in WT SOD1 protein levels from worms co-expressing WT SOD1;torsinA (Fig. 4E,G) when exposed to the proteasome inhibitor (*P*<0.001). Although inhibition of degradation was also detected in the soluble fractions isolated from G85R SOD1 worms co-expressing torsinA (Fig. 4C versus 4G), there was no detectable change in G85R SOD1 protein levels in animals expressing G85R SOD1;torsinA when compared with control, G85R SOD1, animals (Fig. 4E,G) treated with MG132. In contrast, the insoluble fraction isolated from *C. elegans* expressing G85R SOD1;torsinA still displayed a significant decrease of SOD1 protein levels when compared with extracts from G85R SOD1 animals alone, both with (Fig. 4F,G) or without (Fig. 4B,C) blocking proteasome function.

## DISCUSSION

Cellular imbalances in protein homeostasis have a contributing role in many neurological diseases (Chen et al., 2010; Metzger et al., 2008; Nery et al., 2011). Cumulative studies reflect a relationship between ER stress and compromised neuronal function underlying ALS (Atkin et al., 2006; Kanekura et al., 2009; Saxena and Caroni, 2011; Saxena et al., 2009). Recent studies using *C. elegans* have provided insights into the cellular dynamics surrounding activation of the UPR by other ALS-related proteins, including FUS and TDP-43 (Vaccaro et al., 2012; Vaccaro et al., 2013).

Here, we used the mutant SOD1 protein as an example target to investigate the potential application of human torsinA in rescuing ER stress associated with ALS. Mutant G85R SOD1 misfolds, aggregates and interferes with neuronal function (Wang et al., 2009). In turn, accumulation of the misfolded SOD1 at the ER activates the apoptosis signal-regulating kinase 1, ASK1, which induces motor neuron death (Nishitoh et al., 2008). The mutant protein has also been shown to bind to Derlin-1, a component of the retrotranslocon complex, disrupting the proteasomal degradation of terminally misfolded proteins.

Previous studies have shown that torsinA, a uniquely metazoan ER-resident protein with chaperone activity, interacts with Derlin-1 and enhances ERAD of misfolded proteins (Nery et al., 2011). Because it is highly expressed in neurons, we hypothesized that enhancing torsinA activity would attenuate the proteostatic dysfunction in affected neurons. Our data support this hypothesis, because overexpression of torsinA reduced ER stress and improved the locomotion defect (and presynaptic dysfunction) caused by mutant G85R SOD1 pan-neuronal expression in transgenic nematodes. This neuronal rescue is also dependent on the interaction with Derlin-1. Furthermore, torsinA promoted the degradation of SOD1 protein, which supports the functional evidence implicating torsinA in facilitating ERAD of protein substrates, putatively attenuating the phenotypic defects observed in the animal behaviors.

Many compounding factors influence the death of the motor neurons in human and animal models of ALS. The neurotransmission defects observed might be an indirect association with the ER stress induced by mutant SOD1. Nevertheless, torsinA exhibits the capacity to restore protein homeostasis to basal levels. Whether this restoration is directly linked to the neuronal rescue of locomotion defects of the animals remains unknown. However, if ERAD is inhibited and induction of apoptosis occurs, the physiological responses and protein modifications observed in these SOD1 animals provide some indication that these pathways are interconnected in influencing pathology.

This concept is not novel, because Jiou Wang and colleagues have shown that, when the *C. elegans* mutant SOD1 animals have higher levels of aggregation, the locomotive defect is exacerbated (Wang et al., 2009). Many additional studies have described a correlation between protein aggregation and ER stress (Pereira, 2013), including models of neurodegeneration such as in Parkinson’s disease, Alzheimer’s disease and ALS (Jiang et al., 2010; Katayama et al., 2004; Mattson et al., 2001; Nishitoh et al., 2008; Smith et al., 2005).

Because RNAi does not necessarily block protein activity completely, we might not be able to fully appreciate the cooperative role between Derlin-1 and torsinA in this attenuated toxicity. Furthermore, the observation that suppressing Derlin-1 function did not completely abolish torsinA rescue indicates that torsinA might be working in conjunction with other unknown factors to mediate a neuroprotective role. It was initially surprising that suppressing *cup-2* (Derlin-1) did not exacerbate the thrashing defect, because there is evidence that Derlin-1 and mutant SOD1 can interact directly. RNAi of *cup-2* has been shown to induce the UPR and ER stress in *C. elegans* (Schaheen et al., 2009; Ye et al., 2004), corroborating the evidence that Derlin-1 plays a substantial role in ERAD. Our qPCR data did not indicate elevated *cup-2* levels in the G85R SOD1 background (data not shown), but this might be attributed to the mild induction of the ER stress response by G85R SOD1, because previous studies have shown that high levels of ER stress (i.e. high concentrations of tunicamycin) induce upregulation of Derlin-1 (Oda et al., 2006). However, it has been postulated that mutant SOD1 can indirectly interact with Derlin-1 via other ERAD components, such as p97, VIMP and E3 ligases (Nishitoh et al., 2008). Derlin-1 interacts with two proteins, US11 and VIMP/p97, which have luminal and cytosolic functions, respectively. TorsinA also interacts with VIMP and p97 in a Derlin-1-dependent manner (Nery et al., 2011) and we predict that loss of Derlin-1 would cause a loss of interaction with the cytosolic proteins, disrupting the ERAD process. The lack of complete loss of rescue of *cup-2* RNAi in the G85R SOD1;torsinA background could also be attributed to compensatory mechanisms of the additional Derlin ortholog in *C. elegans* (R151.6). There is evidence that the other Derlin proteins (2 and 3) play a role in ERAD in mammalian cells (Oda et al., 2006). Likewise, other cytosolic proteins, such as Ura3p, are regulated by ERAD via the retrotranslocon components for proteasomal degradation (Metzger et al., 2008). Overall, there are numerous proteins involved in the retrotranslocon complex and disruption to any of these would likely enhance the build-up of misfolded proteins.

We propose that the interaction of G85R SOD1 with the retrotranslocon complex might be mediated by torsinA itself. As implicated earlier, chaperones are necessary to hold misfolded proteins in the ER and then transport them to the retrotranslocon machinery for proper transfer to the cytosol (Nakatsukasa and Brodsky, 2008; Nishikawa et al., 2001). Based on our model system in which torsinA is overexpressed, we predict that torsinA would properly direct the aberrant G85R SOD1 protein to the retrotranslocon for proper degradation. Alternatively, torsinA activity might alter the conformation of the retrotranslocon complex via Derlin-1, facilitating more efficient translocation to the cytoplasm for degradation.

The effect of the proteasome-inhibitor-treated animals was insightful and demands further investigation. In this regard, although a 2-fold increase in the WT SOD1 protein levels was observed in the presence of inhibitor and torsinA expression, no significant change between soluble G85R SOD1 levels with or without torsinA was detected. With blocking the proteasome, the impact of torsinA on G85R SOD1 protein levels was therefore abolished. Overall, these data support the hypothesis that torsinA promotes degradation of mutant G85R protein through the proteasome. However, the lack of an observed increase in G85R SOD1 in this experiment is indicative of another mechanism through which the mutant protein is degraded during proteasomal inhibition. Importantly, Li et al. recently demonstrated that mutant SOD1 (G93A) expression in *C. elegans* motor neurons also led to locomotive dysfunction and that upregulation of autophagy rescued the observed motor defects (Li et al., 2013). Likewise, there is evidence that ER stress and the activated UPR can trigger non-proteasomal degradation pathways (Shenkman et al., 2007); this is also apparent in the insoluble fraction of mutant SOD1, where MG-132 treatment did not impact G85R SOD1 protein levels. Therefore, a compensatory interplay between ERAD and autophagic degradation mechanisms might coordinately function to co-regulate the consequences of misfolded SOD1 *in vivo*.

Several significant features were identified in our analysis of the *C. elegans* SOD1 animal model system. First, we were able to recapitulate the induction of ER stress observed in mammalian systems with a mutant form of human SOD1. We also reproduced an *in vitro* characteristic of SOD1 dynamics in *C. elegans* by showing that mutant SOD1 is susceptible to changes in temperature and that it exacerbates its toxic effects at higher temperatures. This occurrence was also depicted in the temperature-dependent response in locomotion (Rodriguez et al., 2002; Tiwari and Hayward, 2005). Furthermore, because aging represents the single greatest risk factor for ALS and other neurodegenerative diseases, it is therefore significant that SOD1 G85R locomotion defects are improved by mutants or RNAi versus select targets in the *daf-2*/insulin-like signaling and aging regulatory pathway of *C. elegans* (Boccitto et al., 2012). Taken together, recapitulation of mammalian G85R SOD1 phenotypic and functional modifying effects in *C. elegans* validates use of the invertebrate ER stress and neuronal models to discern additional mechanistic insights into proteostatic regulation in ALS.

We also demonstrated that animals overexpressing WT SOD1 exhibited mild but significant defects. This phenomenon was specifically discerned in the locomotion assays performed on both solid and liquid media. Notably, WT SOD1 is also associated with ALS, because it has the propensity to misfold and/or lose essential interactions necessary for proper neurotransmission (Gagliardi et al., 2010). When overexpressed, WT SOD1 might also be partly targeted for degradation in a similar fashion as mutant SOD1. Wang and colleagues did not observe such differences between control animals and WT SOD1 (Wang et al., 2009). However, they did not compare the thrashing rates to non-transgenic animals; thus, this phenotypic distinction could have been overlooked. We did not observe this defect in all behavioral responses (i.e. aldicarb), indicating that WT and mutant SOD1 elicit differential toxicities.

Our collective results broadly suggest that increasing levels or activity of torsinA could have a therapeutic benefit for ALS. Using *C. elegans* as a screening platform, we previously identified compounds that subsequently enhanced torsinA-dependent trafficking and alleviated aberrant behavior associated with DYT1 dystonia mice (Cao et al., 2010). Interestingly, the torsinA activators characterized in this prior work were antibiotics of the beta-lactam class (i.e. ampicillin). This is intriguing considering independent studies, unrelated to torsinA biology and presumably mechanistically distinct, resulted in the identification of this same class of compounds as therapeutic candidates used in human clinical trials for ALS (Rothstein et al., 2005). Unfortunately, the robust attenuation of dysfunction in both the locomotion and ER stress assays in the specific worm models utilized here precluded our ability to examine the effects of chemically enhanced torsinA activity in this study. Regardless, we contend that these findings lay the groundwork for further exploration of torsinA-targeted therapeutics in ALS.

## MATERIALS AND METHODS

### Plasmid constructs and transgenic strains

Nematodes were grown and maintained using standard procedures (Brenner, 1974). The starting strains used were SJ4005 *zcIs4*[*hsp-4*::GFP], UA97 *baIn16* [P*_ges-1_*::WT torsinA, *rol-6*] (Chen et al., 2010), P*_snb-1_*::WT SOD1, *nuIs152* [P*_unc-129_*::GFP::*snb-1*] and P*_snb-1_*::G85R SOD1, *nuIs152* [P*unc-129*::GFP::*snb-1*] (Wang et al., 2009).

Human *torsinA* (*DYT1*) and human *SOD1* cDNAs were amplified by PCR to create plasmids using recombinational cloning [Gateway Technology (Invitrogen)]. The K108A mutation *torsinA* clone was provided by Phyllis Hanson (Washington University). The N-terminal 40-amino-acid deletion (Δ1–40) *torsinA* was created as described previously (Chen and Burdette et al., 2010). Using the primers listed below, human *torsinA* variants and *SOD1* were amplified and the products were initially inserted into the pDONR221 entry vector. The *SOD1* constructs were then recombined into the pDEST-JM16 destination vector (Aamodt et al., 1991) and the *torsinA* construct was then recombined into the pDEST-SNB-1 destination vector. All entry and destination vectors were verified by DNA sequencing.

The following primers were used to amplify Flag-tagged WT and G85R *SOD1* as well as Flag-tagged *torsinA*. The lowercase nucleotides represent the Gateway attB sequences. The underlined represents the Flag tag sequence. For RT-PCR analysis, the same Flag-tagged primers for *SOD1* and *torsinA* were used to eliminate the chance of amplifying endogenous mRNA.

Flag-tag *SOD1*: 5′-ggggacaagtttgtacaaaaaagcaggctccATGGACTACAAGGACGACGATGACAAGATGGCGACGAAGGCCGTGTGC-3′ and 5′-ggggaccactttgtacaagaaagctgggtcTTATTGGGCGATCCCAAATTAC-3′.

Flag-tag *torsinA*: 5′-gggacaagtttgtacaaaaaagcaggctccATGGACTACAAGGACGACGATGACAAGATGAAGCTGGGCCGGGCCGTG-3′ and 5′-ggggaccactttgtacaagaaagctgggtcGTTAGATTATTACTACGATGATTGAGGTACCCC-3′.

*cup-2*: 5′-CAAGTTCCAGTTCTGGAGGC-3′ and 5′-CATGCAAAGTCCCGAGCAGAAG-3′.

*ama-1*: 5′-CGAGTCCAACGTACTCTCC-3′ and 5′-GATGTTGGAGAGTACTGAGC-3′.

The P*_ges-1_*::WT *SOD1* and P*_ges-1_*::G85R *SOD1* were co-injected independently into the *hsp-4*::GFP strain at 20 μg/ml each with 50 μg/ml of P*_unc-54_*::mCherry as the injection marker. The resulting stable extrachromosomal transgenic lines were UA217 *baEx130* [P*_ges-1_*::WT SOD1, P*_unc-54_*::mCherry], *zcIs4*[P*_hsp-4_*::GFP]; UA218 *baEx131* [P*_ges-1_*::G85R SOD1, P*_unc-54_*::mCherry], *zcIs4*[P*_hsp-4_*::GFP]. At least three independent lines were created and a representative line utilized in the analyses.

The P*_snb-1_*::torsinA was co-injected with a P*_myo-2_*::mCherry injection marker into N2 worms and the resulting transgenic line was UA220 *baEx132* [P*_snb-1_*::WT torsinA, P*_myo-2_*::mCherry]. At least three stable lines were created and analyzed. Representative lines were selectively integrated by UV irradiation using a Spectroline UV crosslinker at 254 nm with an energy setting of 20 mJ/cm^2^. After integration, each strain was outcrossed at least five times with N2 Bristol to remove potential random mutations generated by UV irradiation. This generated line UA222 *baIn43* [P*_snb-1_*::WT torsinA, P*_myo-2_*::mCherry].

To examine torsinA-mediated rescue of SOD1 (WT or mutant), integrated lines of torsinA [pan-neuronal (*snb-1* promoter) or intestinal] were crossed with the appropriate SOD1 lines, generating the following lines. Intestinal lines: UA239 *baEx130* [P*_ges-1_*::WT SOD1, P*_unc-54_*::mCherry]; *zcIs4*[*hsp-4*::GFP]; *baIn16* [P*_ges-1_*::WT torsinA, *rol-6*] and UA219 *baEx131* [P*_ges-1_*::G85R SOD1, P*_unc-54_*::mCherry]; *zcIs4*[*hsp-4*::GFP]; *baIn16* [P*_ges-1_*::WT torsinA, *rol-6*]. Pan-neuronal lines: UA240 *baIn43* [P*_snb-1_*::WT torsinA, P*_myo-2_*::mCherry]; P*_snb-1_*::WT SOD1, *nuIs152* [P*_unc-129_*::GFP::*snb-1*] and UA224 *baIn43* [P*_snb-1_*::WT torsinA, P*_myo-2_*::mCherry]; P*_snb-1_*::G85R SOD1, *nuIs152* [P*_unc-129_*::GFP::*snb-1*].

To examine torsinA-dependent rescue of G85R-SOD1-induced ER stress, K108A torsinA (P*_ges-1_*::K108A torsinA, 50 ng/μl) or Δ1–40 torsinA (P*_ges-1_*:: Δ1–40 torsinA, 50 ng/μl) was injected into UA218 *baEx131* [P*_ges-1_*::G85R SOD1, P*_unc-54_*::mCherry]; *zcIs4*[*_hsp-4_*::GFP] along with a phenotypic marker *rol-6* (50 ng/μl). Two stable lines were generated for K108A torsinA: UA264-a and b *baEx155*-a and b [P*_ges-1_*::K108A torsinA, *rol-6*]; *baEx*131 [P*_ges-1_*::G85R SOD1, P*_unc-54_*::mCherry], *zcIs4*[P*_hsp-4_*::GFP]. Four stable lines were generated for Δ1–40 torsinA: UA265-a,b,c, and d *baEx156*-a,b,c, and d [P*_ges-1_*::Δ1–40 torsinA, *rol-6*]; *baEx*131 [P*_ges-1_*::G85R SOD1, P*_unc-54_*::mCherry], *zcIs4*[P*_hsp-4_*::GFP].

### Fluorescent analysis of *hsp-4*::GFP worms

ER stress was examined in late L4-stage animals that were transferred manually to NGM plates. Worms were mounted on a 2% agarose pad and analyzed with a CCD camera (Photometrics CoolSnap HQ) on a Nikon E800 microscope at 40× magnification. GFP intensity was measured in pixels and assigned arbitrary units (a.u.) from a 100×100 μm region of the anterior-most region of the intestine, directly behind the pharynx of each animal using MetaMorph software (Molecular Devices Corp., Sunnyvale, CA). For each strain and condition, at least 30 animals were quantitated in three independent replicates. Comparisons were done on GFP pixel intensity between all strains expressing SOD1 and/or GFP unless otherwise noted. For each assay, the data presented are the normalized fold change mean ± s.d. of three independent trials for every strain or condition unless otherwise noted. Each trial consists of the GFP intensity from 30 animals averaged. Normalization compares GFP intensity for all the samples (*hsp-4*::GFP alone or with torsinA and/or SOD1) divided by the average of *hsp-4*::GFP alone (at their respective temperature).

### RNA extraction, cDNA preparation and quantitative RT-PCR

For each independent sample, total RNA was isolated from 100 late L4 to young adult hermaphrodite worms, as previously described (Hamamichi et al., 2008). The worms were washed three times in M9 buffer to remove bacteria and then kept in 30 μl of water and frozen at −80°C for at least 1 hour. After thawing, 500 μl of TRI reagent (Molecular Research Center) was added to the samples and incubated for 10 minutes at room temperature. The samples were freeze-thawed four times in liquid N_2_, vortex for 15 seconds with 50 μl of 1-bromo-3-chloropropane (Acros Organics), incubated for 10 minutes at room temperature, and centrifuged for 15 minutes at 19,745 ***g*** at 4°C. The supernatant was transferred to an RNase-free tube, mixed with 1.5 μl of glycoblue (Ambion) and 250 μl of −20°C pre-chilled isopropanol, and stored overnight at −20°C. The next day, the sample was centrifuged for 15 minutes at 19,745 ***g*** at 4°C and the supernatant was discarded. The pellet was washed with 200 μl of RNase-free ethanol (75%) and resuspended in 10 μl of DEPC-treated water. The genomic DNA contamination was removed with 1 μl of DNase I (Promega) treatment for 15 minutes at 37°C, then with DNase Stop for 10 minutes at 65°C. cDNA was synthesized with iScript Reverse Transcription Supermix for RT-qPCR (Bio-Rad) following the manufacturer’s protocol.

Quantitative real-time PCR reactions were performed using IQ SYBR Green Supermix (Bio-Rad) with the CFX96 Real-Time System (Bio-Rad). Each reaction contained: 7.5 μl of the IQ SYBR Green Supermix, 200 nM of forward and reverse primers, and 0.3 μl of cDNA, to a final volume of 15 μl. The cycling conditions were as follows: polymerase activation and DNA denaturation at 95°C for 3 minutes, followed by 40 cycles of 10 seconds at 95°C, 30 seconds at 60°C. After the final cycle, a melting curve analysis was performed using the default setting of CFX96 Real-Time System. A single melt peak for each targeted gene was observed and no non-specific amplification was detected in each reaction mixture by agarose gel electrophoresis.

Primer sequences used for quantitative RT-PCR: for *cup-2*: full-length gene sequence was obtained from WormBase and primers were designed by the Primer3 software and potential secondary structures of the amplicon were evaluated by MFOLD software. MFOLD analysis was performed using default settings and 50 mM Na^+^, 3 mM Mg^2+^ and 60°C.

For spliced *xbp-1*, *tba-1* and *act-1*: primer sequences were as previously described (Richardson et al., 2010). *cup-2* forward: 5′-ACATATCGCGGAAGATCAGC-3′; *cup-2* reverse: 5′-ATCGCATTCCAAACCAGAAC-3′; spliced *xbp-1* (R74.3a) forward: 5′-TGCCTTTGAATCAGCAGTGG-3′; spliced *xbp-1* (R74.3a) reverse: 5′-ACCGTCTGCTCCTTCCTCAATG-3′; *act-1* (T04C12.6) forward: 5′-CTTGGGTATGGAGTCCGCC-3′; *act-1* (T04C12.6) reverse: 5′-TTAGAAGCACTTGCGGTGAAC-3′; *tba-1* (F26E4.8) forward: 5′-GTACACTCCACTGATCTCTGCTGACAAG-3′; *tba-1* (F26E4.8) reverse: 5′-CTCTGTACAAGAGGCAAACAGCCATG-3′.

PCR efficiency was calculated from standard curves that were generated using serial dilutions of a cDNA pool of all worm samples. *act-1* (*E*=88.2%, *R*^2^=0.997, slope=−3.641). *tba-1* (*E*=94.2%, *R*^2^=0.999, slope=−3.469). Spliced *xbp-1* (*E*=89.9%, *R*^2^=0.994, slope=−3.592). *cup-2* (*E*=99.1, *R*^2^=0.998, slope=−3.343).

All targeted genes were measured in triplicate and three independent biological replicates were tested for each sample. No amplification was detected in NTC and NRT controls. The Cq values recorded by CFX Manager Software version 3.0 (Bio-Rad) were exported into qBase^PLUS^ version 2.6 (Biogazelle) for determining reference target stability (GeNorm M <0.5, CV <0.2) and relative expression, which was normalized to the mRNA level of *tba-1* and *act-1*. The change of target gene expression was considered significant when *P*<0.05 was calculated by one-way ANOVA with Tukey post-hoc test.

### Locomotion (plate) assay

For the locomotion analyses, strains were compared by evaluating speed of movement. Animals were video recorded and, using the Worm Tracker established by Ramot and colleagues (Ramot et al., 2008), the average speed was calculated. The data presented represents the normalized speed for all the samples divided by the average observed in N2 controls alone and are represented as fold change mean ± s.d. of three independent trials for every strain or temperature condition. Each trial consists of the speed from 20 animals averaged. Statistics were performed using Student’s *t*-test.

### Liquid thrashing assay

Animals that were cultivated at 20°C were placed in a drop of water, allowed to acclimate for 1 minute and the animals were video recorded for 1 minute. The videos were reviewed and the thrashing rates were counted. At least 30 animals were tested for each transgenic line and replicated three times. Animals were analyzed at day 4, 6 and 8 post-hatching. Only day 6 is represented but the results of significant rescue by torsinA were the same for all days of analysis. Error bars represent s.e.m.; statistics were performed using Student’s *t*-test.

### Western blotting

Animals were cultivated at 20°C until they reached the adult gravid stage and then bleached to synchronize the population. The embryos were allowed to develop on NGM plates seeded with OP50 bacteria. For proteasome inhibitor treatment, embryos isolated from bleached gravid adults were placed on NGM plates that contained 50 μM MG-132 incorporated into the media. Once the animals reached the L4 larval stage, they were washed with water and placed in a 1.5 ml microcentrifuge tube. The final volume of concentrated animals was 100 μl. Animals initially underwent three freeze-thaw sessions in liquid nitrogen to break open the cuticle. 300 μl of RIPA buffer (150 mM NaCl, 50 mM Tris pH 8.0, 1 mM EGTA, 5 mM EDTA, 1% NP40, 0.5% sodium deoxycholate, 0.1% SDS) and complete protease inhibitor cocktail was added. Animals were sonicated several times followed by a soft spin to remove large debris. The solution was then centrifuged at 16,000 ***g*** for 3 hours to isolate the soluble and insoluble fractions. The pellet was rinsed several times with RIPA buffer to remove any residual supernatant. Protein concentration was determined by the BCA assay and equal protein concentrations were run on SDS-PAGE gels under reducing conditions and probed with anti-SOD1 antibody (Cell Signaling #2770) and anti-actin (MP #691001). Blots were developed using the Fujifilm Image Reader LAS-4000. Multigage software v3.0 (Fujifilm) was used to quantify protein levels.

### RNA quantitation

To detect mRNA of transgenic animals through semi-quantitative RT-PCR, the same procedure for isolating RNA was performed as described previously (Locke et al., 2006). Primers were designed to be specific to human *torsinA* and *SOD1*. The housekeeping gene *ama-1*, encoding RNA polymerase II, was used as a baseline control. The PCR-amplified products were separated on a 0.8% agarose gel and visualized by GelRed (Biotium).
